# Bionic Multi-Legged Robots with Flexible Bodies: Design, Motion, and Control

**DOI:** 10.3390/biomimetics9100628

**Published:** 2024-10-15

**Authors:** Xiang Li, Zhe Suo, Dan Liu, Jianfeng Liu, Wenqing Tian, Jixin Wang, Jianhua Wang

**Affiliations:** 1 Key Laboratory of CNC Equipment Reliability, Ministry of Education, School of Mechanical and Aerospace Engineering, Jilin University, Changchun 130022, China; lixiang_sub@163.com (X.L.); suozhe23@mails.jlu.edu.cn (Z.S.); liujf23@mails.jlu.edu.cn (J.L.); 2 National Key Laboratory of Special Vehicle Design and Manufacturing Integration Technology, Inner Mongolia First Machinery Group Co., Ltd., Baotou 014030, China; liudan198965112@163.com; 3 FAW Tooling Die Manufacturing Corporation, China FAW Group Co., Ltd., Changchun 130013, China; tianwenqing83@163.com; 4 College of Automotive Engineering, Jilin University, Changchun 130022, China

**Keywords:** bionic robots, multi-legged robots, flexible body, spine, CPG, bionic control, locomotion

## Abstract

Bionic multi-legged robots with flexible bodies embody human ingenuity in imitating, learning, and exploring the natural world. In contrast to rigid-body robots, these robots with flexible bodies exhibit superior locomotive capabilities. The flexible body of the robot not only boosts the moving speed and walking stability but also enhances adaptability across complex terrains. This article focuses on the innovative design of flexible bodies. Firstly, the structural designs, including artificial spines and single/multi-axis articulation mechanisms, are outlined systematically. Secondly, the enhancement of robotic motion by flexible bodies is reviewed, examining the impact that body degrees of freedom, stiffness, and coordinated control between the body and limbs have on robotic motion. Thirdly, existing robotic control methods, organized by control architectures, are comprehensively overviewed in this article. Finally, the application prospects of bionic multi-legged robots with flexible bodies are offered, and the challenges that may arise in their future development are listed. This article aims to serve as a reference for bionic robot research.

## 1. Introduction

The vast array of animals in nature has served as an inexhaustible source of inspiration for the design of robots. Cheetahs exhibit breathtaking speed, with their exceptional limb coordination and flexible spines, while reptiles, such as crocodiles, despite their relatively short limbs, achieve both rapid and stable movement through skillful lateral body movements, even possessing amphibious capabilities. Cockroaches, on the other hand, navigate effortlessly through complex environments, with their remarkable flexibility in the limbs and body. Creating robots that can mimic the flexible movements of these animals has long been a lofty aspiration of human technological exploration.

**Figure 1:**
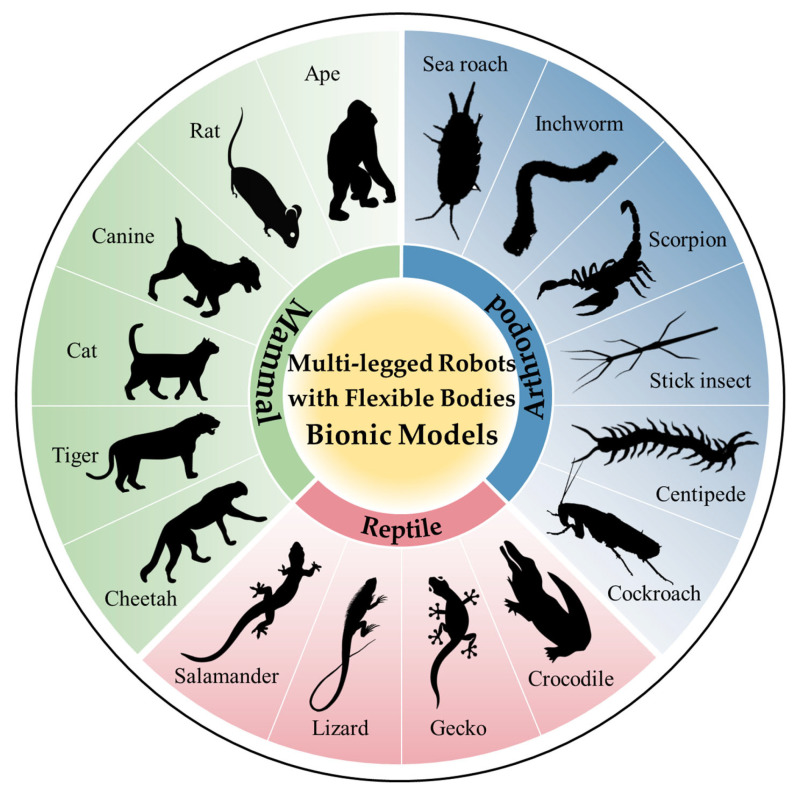
Bionic models of multi-legged robots with flexible bodies.

To better capture the essence of animal motion in nature, researchers have built upon traditional rigid-body robot technology, delving deeper into the design of flexible bodies, and recognizing the crucial role that body motion plays in the overall performance of robots. This shift has led to the emergence of remarkable creations, such as the cheetah robot Cheetah-I [1], the lizard robots ELIRO-I/II [2,3], and the cockroach robot AMOS-II [4], all of which possess highly flexible bodies capable of performing a range of biomimetic movements that traditional rigid-body robots struggle with. Additionally, animals such as salamanders and centipedes have also become sources of inspiration for bionic design, with scientists using these bionic robots to delve into the mysteries of biological motion. Figure 1 presents a list of documented bionic models of robots.

**Figure 2:**
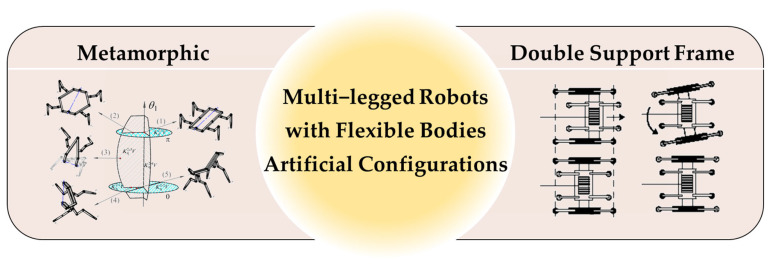
Artificial configurations of robots with flexible bodies.

Beyond the inspiration drawn from nature, humans are also striving to develop robots with even more flexible movement mechanisms. For instance, the Origaker robot [5] achieved morphological transformations in its body through innovative metamorphic mechanisms, while the MELCRAB-2 robot [6] combined a double-support frame with a reciprocating mechanism to enhance its walking flexibility, as depicted in Figure 2.

The flexible body has a profound impact on the robot motion performance: it not only significantly improves the walking speed and stability but also greatly enhances the robot’s adaptability to complex terrains. This article focuses on multi-legged bionic robots with flexible bodies (excluding bipedal humanoid robots and fully soft robots), systematically reviewing the body structural forms of these robots (Section 2), summarizing the positive effects of flexible bodies on robot motion performance (Section 3), and outlining control methods for robots with flexible bodies (Section 4). Finally, the article looks ahead to the application prospects and remaining challenges for the development of bionic robots (Section 5).

## 2. Ways to Achieve Flexible Bodies 

**Figure 3:**
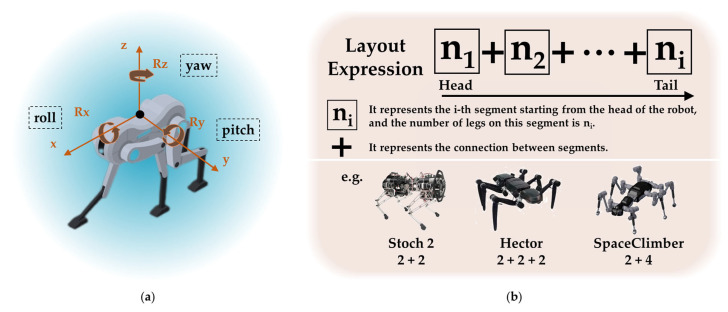
(**a**) The coordinate system of the robot. (**b**) The robot structural layout expression, and examples: Stoch 2 consists of two segments, with two legs on each segment [7]. Hector is composed of three segments, with two legs on each segment [8]. SpaceClimber is comprised of two segments, with two legs on the head segment and four legs on the tail segment [9].

Compared with rigid-body robots, robots with flexible bodies have the most significant structural feature, which is the existence of multiple body segments, which are connected through various ways, realizing the flexibility of the robot body. This article summarizes the main connection methods for achieving body flexibility: artificial spine, single-axis or multi-axis articulation, soft structure, metamorphic structure, etc. To describe the DOFs (degrees of freedom) of the flexible body and the structural characteristics of the robot, this article defines the robot coordinate system and robot structural layout expression, as shown in Figure 3.

### 2.1. Artificial Spine

Artificial spines are mainly applied to quadruped robots that mimic mammals. The artificial spine is mainly composed of a support element, an elastic element, and a driving element (there are also compliant artificial spines that do not have driving elements, such as Cheetah-I [1], which mainly rely on hind legs to drive the spine). The support element, equivalent to a set of vertebrae, is used to connect the front and rear segments of the robot and forms the rotary joint for the curvature of the spine. The elastic element is set between support elements or in parallel with the entire support structure between the front and rear segments of the robot, serving to limit the range of spine motion and buffer, reset, and store energy. The driving element prompts the spine to bend, enabling the robot to acquire a flexible body and achieve more mobility. In this paper, artificial spines are classified into two categories: active spines and compliant spines. Active spines possess the four elements, enabling robots to independently control the bending of the spine through driving elements. In contrast, compliant spines lack these driving elements, and the bending of the spine is passively altered by the movement of the limbs and changes in terrain, with the robot unable to independently control the spine bending angle. In addition, compliant spines can only maintain morphology via the elasticity of the materials.

#### 2.1.1. Active Spine

Both Kitty [10] and Transleg [11,12] employed rigid materials to serve as vertebrae, which are connected by ball joints. Silicon blocks are filled between the vertebrae to simulate the lumbar discs. The cables pass through the vertebrae and silicon blocks, arranged symmetrically on the upper, lower, left, and right sides of the artificial spine. These cables are pulled by motors located on the front and rear body segments to achieve the flexion of the spine. Canid [13,14] is a robot modified based on RHEX, with leaf springs connecting the front and rear body segments and supported by a set of vertebral brackets made of carbon fiber. Two sets of parallel cables, one above and one below, pass through vertebral brackets, and the cables can drive the spine to bend in the sagittal plane. Also, Inu [15] adopted a similar artificial spinal structure. The artificial spine of NeRmo [16] mimicked the physical structure of a biological spine, utilizing the tensile strength and flexibility of nylon to bear its weight. The spine consists of two parts: the front section is rigid and responsible for accommodating all electronic equipment, while the rear section imitates vertebrae by alternately stacking four lumbar vertebrae and lateral flexion joints. The robot in [17] used an inflatable rubber tube to fill the spine, and the spine was also driven by cables. QuaDRoPECS [18] utilized a continuous elastomer made of fiber-reinforced plastic (FRP) plates, which serve both as a support element and an elastic element. Two sets of elastomers are horizontally arranged between the front and rear body segments on the left and right sides, and they are driven by motors to deform the elastomers through pulling cables. Cheetah-Cub-S [19] utilized leaf springs made of polyoxymethylene (POM), serving both as a support element and an elastic element. The center of the spine is a servo motor that pulls cables to drive the bending of the leaf springs. The leaf springs connect the spine motor to the two body segments. To reduce externally induced torsion (Rx), two leaf springs are installed in parallel, one above the other. In subsequent improvements, the cable drive was replaced with a lever drive. The artificial spine of Laika [20] is composed of five tetrahedral vertebrae made of rubber. The vertebrae are connected through a tensegrity structure. The bending of the spine is achieved by pulling parallel cables arranged vertically and horizontally, enabling flexibility in three directions: Rz, Ry, and Rx. The robots in [21,22,23] all adopted an artificial spine structure consisting of vertebral bodies and intervertebral discs. They utilized pneumatic muscles to drive the spine, and at the same time, the pneumatic muscles can also adjust the stiffness of the spine. Lynx SV2 and Lynx SV3 [24] are composed of 3D-printed “vertebrae” that are connected in series through pins to link the front and rear segments, forming a multi-joint spine. The cables can be shortened to reduce the distance between the front and rear segments, thus achieving active flexion of the spine. Parallel glass fiber rods serve as elastic elements, allowing the spine to passively return from flexion to its original position. Compared to SV2, SV3 employed two glass fiber rods as flexible elements. The main differences lie in the stiffness of the spine and the connection position with the rear segment. Both SV2 and SV3 require pre-tensioning of their elastic elements to maintain tension in the cables when in a standing position.

#### 2.1.2. Compliant Spine

The spine of Cheetah-I [1,25] is composed of polyurethane rubber rings sandwiched between hard plastic vertebrae. A differential spine drive in the hind legs couples the hind leg movement with the spinal motion, enabling the spine to store and release potential energy during movement. When both hind legs move forward simultaneously, the spine bends; when the legs move backward, it extends. The polyurethane rubber rings can store elastic energy and return it during the galloping. During walking or trotting, when the left and right legs are not synchronized, the spine moves very little, remaining mostly stationary. The design of Cheetah-I emphasizes bio-inspired structures, accurately mimicking the biomechanical characteristics of real cheetahs, and is primarily used for in-depth research in biomechanics and kinematics. However, the artificial spine was eliminated in the subsequent Cheetah-II [26]. The design philosophy of Cheetah-II has shifted, focusing more on practical applications. By eliminating the spinal structure, the control system of Cheetah-II becomes simpler and more efficient, while also significantly enhancing its loading capacity.

The compliant spine of LittleApe [27] used silicone rubber for the intervertebral discs. Bowden cables connect the artificial vertebrae and intervertebral discs, and the bending and twisting behavior of the spine is influenced by the stiffness of the intervertebral discs and the tension of cable. A similar approach was also employed in the robot mentioned in [28]. Both Tiger [29] and Fanari [30] featured compliant spines. Tiger used thin-metal sheets as waistlines to connect both segments. To limit the downward bending of the spine in the sagittal plane, Tiger installed some lightweight segments with very small horizontal gaps at the top of the sheet. The gaps between the lightweight segments control the range of bending. To create spine elasticity, a spring is connected in parallel to the artificial spine between the segments, making its stiffness adjustable. The spring is also used to store energy. Fanari is capable of bidirectional bending in the sagittal plane. It has 13 pieces of plexiglass arranged outside the metal sheet to limit the bending direction of the spine. The gaps between the plexiglass pieces are used to adjust the bending range. Four sets of springs are installed on the outer side of the spine to achieve elasticity, and three pairs of springs are added on the inner side of the spine.

**Figure 4:**
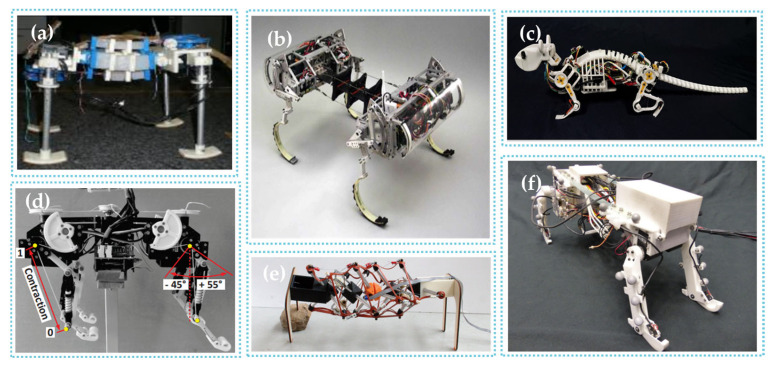
Some typical robots with artificial spines. (**a**) Kitty [31], (**b**) Canid [32], (**c**) NeRmo [33], (**d**) Cheetah-Cub-S [19], (**e**) Laika [20], and (**f**) QuaDRoPECS [18].

Figure 4 shows some typical robots with artificial spines.

### 2.2. Single-Axis or Multi-Axis Articulation

The robot connects each segment through articulation, thus obtaining the freedom of the body in one or more directions, and the body movement has a distinct axis of rotation. The segments of the robot can be articulated either rigidly or using series or parallel springs. Most of the robot articulated axes are active and directly driven by motors (the robots in [34,35] adopted linkage-driven mechanisms), but there are also a small number of compliant joints connected with springs to reset the body.

**Figure 5:**
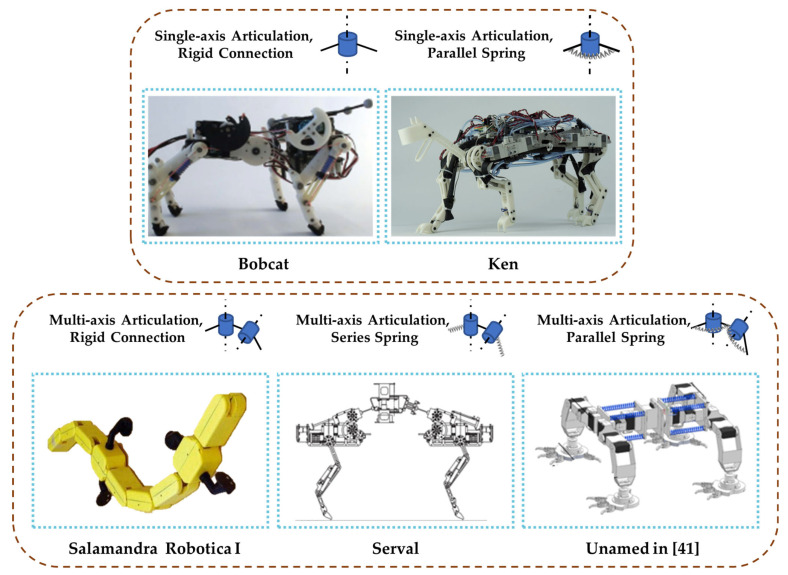
Five articulation types for robot segments: Bobcat [38], Ken [39], and Salamandra Robotica I [40], with permission from AAAS, Serval [37], and unnamed robot in [41].

Figure 5 shows several types of robots with articulated joints. “Single-axis articulation” refers to only one articulated axis between two body segments of a robot, allowing the robot body to achieve freedom of movement in one direction, such as the Salamandra Robotica I [36]. “Multi-axis articulation” indicates that there are multiple articulated axes between two body segments of a robot, enabling the robot body to achieve multiple freedoms of movement in different directions, such as the Serval [37] and the WR series of bionic rat robots.

Table 1 summarizes the advantages and disadvantages of active spine, compliant spine, single-axis articulation, and multi-axis articulation.

### 2.3. Other Ways to Achieve Flexible Bodies 

“Artificial spine” and “single-axis or multi-axis articulation” are the two most common approaches to achieving flexibility in a robot body. Besides these, some other ingenious designs can give the robot body additional DOFs.

Charlie [42] and ParaWalker [43] both utilized a 6-DOF Stewart Platform [44] to connect the two segments of the robot, enabling omnidirectional movement for the robot body. LightDog [45] employed an integrated elastic material design for the flexible passive spine. Similarly, the authors of [46] also used elastic material for the spine but incorporated McKibben actuators to make it active. The authors of [47] utilized a piezoelectric material as the robot spine, which bends and stretches under the influence of an electric field, enabling rapid movement of insect-sized robots. The Origaker series of robots [48,49] utilized the transformative capability of mechanisms through metamorphic mechanisms composed of multiple links [50,51]. This approach allows planning of the robot morphological changes [48], enabling transitions between the forms of reptiles, mammals, and arthropods. Renny [52] connected its two body segments through spherical joints and used pneumatic biomimetic muscles to drive the movement of the body. The inchworm-mimicking robot in [53] employed an inflatable and differentially actuated Octagon-origami structure as its body, enabling flexible movement. The authors of [54] proposed a soft actuator with a tunable bistable spine mechanism for rapid energy storage or release, allowing the robot to achieve fast movements. The Hector [8] biomimetic stick insect robot employed a 2-DOF Spindle Joint between its segments, enabling relative movement in the Rz, Ry, and X directions between body segments. The Octopod robot in [55] utilized a Bricard linkage (a spatial six-bar closed-chain mechanism) to connect the front and rear body segments, while the biomimetic centipede robot in [56] connected its segments using Sarrus linkages.

Danta-II [57], MELCRAB-2 [6], and robots in [58,59] are six-legged robots with a “double-support-frame” configuration. Each frame represents a stable three-legged support, and the robots achieve forward locomotion through alternating support frames via reciprocating mechanisms. Similarly, ParaWalker [43] is also a six-legged robot with a “double-support-frame” configuration, but it employed a Stewart Platform with more DOFs as its reciprocating mechanism.

**Figure 6:**
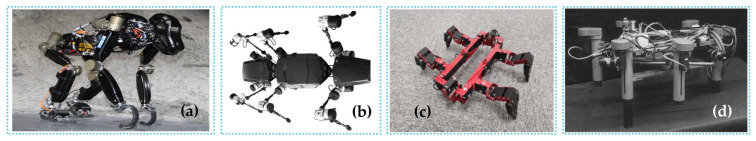
Other designs for robots with flexible bodies: (**a**) Charlie [60], (**b**) Hector [8], (**c**) Origaker [48], and (**d**) unnamed robot in [58].

Figure 6 shows some of the robots with flexible bodies.

At the end of this section, this article reviews some of the body-flexible robots documented in the retrievable literature, summarizing their ways to achieve body flexibility, the additional DOF the robot body gained, the robot’s structural layout, and their bionic models, as shown in Table 2 and Table 3.

Table 2 mainly focuses on quadruped robots with flexible bodies, while Table 3 reviews robots with more than four legs. Both Table 2 and Table 3 are arranged in alphabetical order by the robots’ names.

## 3. Motions Generated by a Flexible Body

Through the above design approaches, the bionic robots have acquired flexible bodies, enabling them to achieve more rich and natural motions.

### 3.1. Motion Enhancement Due to the Flexible Body

#### 3.1.1. Motions in the Sagittal Plane: Bounding and Galloping

**Figure 7:**
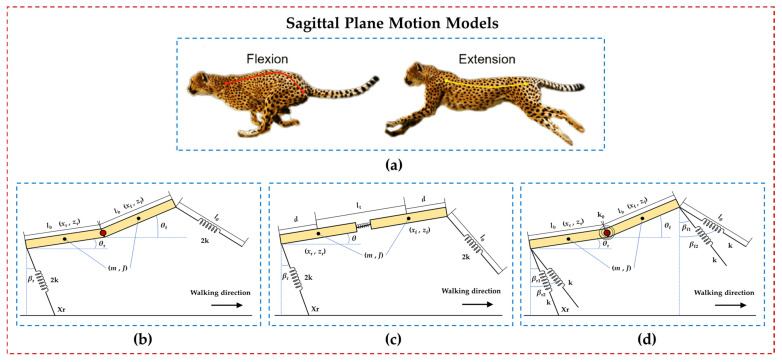
Sagittal plan motion models for bounding and galloping. (**a**) The spinal motion of a cheetah while it is galloping (figure captured from [54]). (**b**) Motion model with single-axis articulation. (**c**) Motion model with linear spring. (**d**) Motion model for asymmetric gait.

Bounding and galloping gaits are the preferred rapid gaits for robots that mimic mammals, especially cheetah bionic robots (Figure 7a). The authors of [98,99,100,101,102,103,104,105,106,107,108,109,110,111] have all established sagittal plane motion models for robots with articulated flexible bodies (such as in Figure 7b), investigating the influence of the system-inherent dynamic characteristics on motion. They revealed how to utilize inherent properties to achieve continuous and stable bounding motion, providing a basis for the setting of mechanical structure parameters and the research of control algorithms for robots.

The authors of [107] provided theoretical evidence that suggested that compared to robots with rigid bodies, the active movement of the body helps to increase the bounding stride length, resulting in enhanced horizontal velocity.

By fitting empirical data, a reduced-order dynamic model was proposed to describe the robot center of mass trajectory during bounding motion. Experiments conducted on the Inu robot demonstrated that body movements can expand the robot workspace, enhance leg ground reaction forces, and improve motion stability [98].

In [99], the robot body was abstracted as parallel springs and dampers, leading to the development of a compliant simplified sagittal plane model. This model explored the relationship between optimal body stiffness, robot speed, and total mass, suggesting the existence of an optimal trunk stiffness at a given speed for optimal energy efficiency.

A reduced-order sagittal plane model with a flexible body was established, connecting front and rear body segments via massless pins and incorporating parallel springs at the hinge points [72,99,108]. The robot legs were simplified as massless springs. Analysis revealed that body movements reduced peak leg forces. When body stiffness was greater during extension than during flexion, the motion more closely resembled that of real quadrupeds. A specific combination of body and leg stiffness enabled stable and continuous jumping. The Kitty robot [31] validated these findings.

Quadruped models with varying numbers of body joints were developed to test the biological concept of the “spinal engine” [112]. The results indicated that a model with two active body rotary joints is enough to mimic the motion behaviors driven by biological spines [100,101].

The authors of [106,109] utilized linear springs to connect the front and rear segments of the robot, establishing a sagittal plane motion analysis model (Figure 7c). Analysis showed that elastic body translation enables the robot to achieve larger strides and initial acceleration.

Quadrupeds tend to adopt symmetric gaits at low speeds and asymmetric gaits at high speeds. The authors of [110,111] established a sagittal plane dynamic model (Figure 7d) capable of analyzing asymmetric gait motions (such as rotary galloping gait), investigating the energy benefits and dynamic stability of articulated flexible bodies during locomotion.

Experiments with robots such as Transleg [12], Kitty [31], Renny [52], Canid [13], and Sugoi-Neco [75] also demonstrated that bounding motions in the sagittal plane, facilitated by the flexible body, enhanced stride length, stability, and energy efficiency in robotic locomotion.

#### 3.1.2. Motions in the Horizontal Plane: Crawling, Trotting, and Turning

The bodies of reptiles, such as geckos and salamanders, primarily undergo bending and undulation motions in the horizontal plane, with a limited motion range in the sagittal plane [113]. As a result, robots designed to mimic these reptiles typically have their bodies equipped with DOFs only in the Rz direction. The bending and undulation of the body in the horizontal plane, coupled with the robot leg extension and stepping, form a zigzag crawling locomotion [36,40,114]. When the wave motion of the body transitions from a stationary wave to a traveling wave, this enables amphibious crawling robots to switch from terrestrial locomotion mode to aquatic locomotion mode [115,116].

For robots mimicking mammals, lateral flexion of the body contributes to increasing the step length in the trotting gait and achieving higher speeds [117]. It also expands the leg workspace, enhancing the robot’s adaptability to complex environments [118]. The body torsion in the horizontal plane adjusts the robot’s center of mass, keeping it within a stable polygonal support area, improving stability margins during locomotion, and maintaining overall balance [119]. Therefore, many disabled quadrupedal animals can still stand or walk even with only three limbs.

Active body bending enhances the robot’s turning capabilities [19]. During in-place turning, body twisting avoids critical stability conditions, improving stability during turning [120]. Researchers utilized NeRmo [16] to validate three turning strategies: using only legs, using only the body, and a hybrid approach combining both the legs and body. The results showed that turning solely with the body was the fastest, while the hybrid method produced a smaller turning radius. Consequently, robots capable of body bending can navigate narrower right-angle bends and S-shaped turns more effectively.

#### 3.1.3. Special Motions

- Climbing

For robots that mimic insects, the bending of the body in the sagittal plane can enhance their ability to climb over obstacles.

Researchers observed cockroaches during climbing and embedded a sensor-driven neural control into the AMOS II robot [121], enabling it to achieve adaptive obstacle climbing capabilities. The Climbing Mini-Whegs^TM^ B31 [95] (where B31 refers to the position of the hinge axis being 31% of the length from the middle leg to the front leg) is an improvement upon the cockroach-inspired Mini-Whegs robot [122]. It incorporated an additional articulated axis into the body, allowing it to bend in the sagittal plane. The influence of the axis position on the climbing ability was studied, and modifications were made to the axis position and range of rotation angles, resulting in the B00 design with enhanced climbing capabilities.

- Animal Behavior

The WR series robots [82,83,84,123,124,125] and SQuRo [74,126] robots were designed concerning the body structure of rats, and their overall size closely resembles that of actual rats. Through the flexible articulated robot bodies, they can perform rat-like actions, such as rearing, mounting, rotating, and U-turns, enabling interactions between robots and rats, thus achieving repeatability in animal social behavior experiments. The research on this series of robots integrated a rat-like robot with actual rats to gain a deeper understanding of animal behavior and potentially find applications in the treatment of psychiatric disorders and the screening of psychotropic drugs in the fields of psychology and pharmacology.

- Unnatural Motions

**Figure 8:**
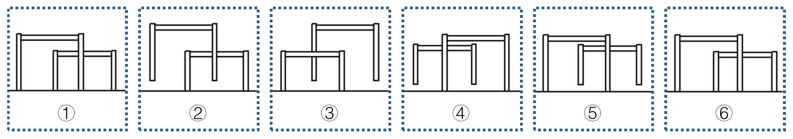
Discontinuous walking motion sequence. The numbers 1−6 in the figure indicate the sequence of discontinuous walking motion.

Robots with double-support-frame artificial configurations, such as Danta-II and MELCRAB-2 [6,43,58,59,127], achieved discontinuous walking motion through alternating advancement of two stably supported frames, as shown in Figure 8. Among them, the authors of [6] employed a gravitationally decoupled length-adjustable leg, enabling the robot to adapt to rugged terrains. MELCRAB-2 [43] further demonstrated a capability akin to “leg–arm coupling”, where one frame can stand and support while the other frame can grip tools for construction work. These non-bionic robots exhibit limited and monotonous locomotion gaits, leading to unnatural behaviors. However, robot turning and pivoting in place are relatively easy and convenient for manual control.

### 3.2. Factors That Influence the Robot’s Motion

- DOF of the body

The DOF of the flexible body is the most important factor affecting the movement of robots. Table 4 summarizes the impact of each DOF on the robot’s motions.

Regardless of the methods of connection among robot segments, the yaw motion of the body can enable the robot to achieve greater speed, walking stability margin, and a smaller turning radius. The advantage of pitch motion in energy efficiency can only be observed in robots with artificial spines. The roll motion of the body enables the robots to have better terrain adaptability, especially the SQuRo-S [74], which can rotate its body segment by 90 degrees to pass through narrow gaps. On the other hand, Laika [20] and CkBot [87] do not have joint-driven legs, so they need to use roll motion to lift and lower their foot ends. Additionally, roll motion also allows robots to recover from falls and regain standing positions. For inchworm robots, body extension facilitates forward movement.

- Stiffness of artificial spine (body-bending angle)

The stiffness of the body is crucial to a flexible-body robot. Excessive body stiffness turns the robot closer to a rigid-body robot, while insufficient stiffness destabilizes the robot motion:

1. If the stiffness of the spine lower side is lower than the upper side, the robot movement becomes more natural [31,52].
2. For a specific robot, there exists an optimal stiffness value that allows the robot to strike a balance between fast motion and energy efficiency.

In the case of robots with articulated bodies, the bending angle of the body influences the robot’s mobility. Similarly, there is an appropriate body-bending angle that enables a robot to achieve a balance between rapid movement and a high stability margin.

- Coordinated actuation

The motion of a robot is a coupling of body movement and limb movement, and the coordinated actuation of the body and limbs exerts a significant influence on the robot’s motion. Whether it is a reptilian robot or a mammalian robot, the oscillation phase between the body-bending motion and the limb-swing motion affects the workspace. When the body is bent to its maximum, the limbs reach their maximum stride amplitude, enabling the maximum step length.

The oscillating phase between the body segments of the salamander robots [36,116] influences the form of body undulation. When there is no phase difference in the oscillation between body segments, the robot body exhibits standing-wave oscillation, suitable for terrestrial locomotion mode; whereas, when there is a phase difference in the oscillation between body segments, the robot body displays traveling-wave oscillation, which is conducive to aquatic swimming mode.

The traveling-wave oscillation of the body in the multi-legged centipede robot [87,93] is influenced by the oscillating phase difference between the robot segments, which in turn affects its walking speed and stability.

## 4. Control of Flexible-Body Robots

There have already been many different methods to control the robots, which can be categorized into three types, as presented in [90]. The first is the precise physical model-based control method, the second is the biologically inspired control method, and the third is the adaptive control method developed using learning algorithms. This article classifies and organizes the control methods for robots with flexible bodies based on their control architectures.

### 4.1. No Control

Fanari [30] is a completely unpowered and uncontrolled robot designed to study the impact of body stiffness on the stability of rapid walking. It is placed on a slope and relies solely on gravity to achieve galloping motion.

### 4.2. Routine Control Methods

#### 4.2.1. Open-Loop Control

Some small robots are only used for qualitative research on the influence of flexible-body movement on robot motions (verify the biological “spinal engine” hypothesis; compared with rigid-body robots, identify the advantages of flexible-body robots), and there is no excessive requirement for control accuracy, so open-loop control is adopted. They usually generate stable rhythm drive by central pattern generators (CPGs) without any sensory feedback, thus forming several fixed patterns of gait. Early flexible-body robots mostly utilized open-loop control, which will not be elaborated further here.

#### 4.2.2. Simple Closed-Loop Control

Based on open-loop control, some robots have introduced sensors and thus acquired sensory feedback, forming a simple closed-loop control. The WR-3 [84,128] achieved precise control over its motion position through closed-loop control of the rotational speed and current of its drive motors. The OSCILLEX [68] employed CPG control with foot-end force feedback, adjusting the oscillation phases of each actuator to coordinate the movements between the robot legs. For non-biomimetic robots, such as ParaWalker [43], precise physical models are used to control system parameters, and force-position feedback is utilized to achieve precise control over the landing position and force distribution during support.

#### 4.2.3. Distributed Control

The distributed control of flexible-body robots comprises a body controller and limb controllers. The body controller governs the bending of the body and can sense the robot’s posture. For multi-segment robots that mimic reptiles and arthropods (such as Pleurobot [129] and the legged swarm system [130]), the body controller regulates the oscillation phases between segments, enabling the alteration of the body oscillation patterns (traveling waves or standing waves). The limb controllers generate the trajectories for the legs and feet. Equipped with joint position feedback and force feedback mechanisms, these controllers can precisely control and adjust the foot placement, detect whether the foot end has encountered an obstacle or missed a step, and enable the robot to perform simple adaptive reflex movements. Furthermore, the limb controllers also coordinate the movements among limbs, preventing interference between them and ensuring that the robot has enough limb support to maintain stability and prevent falling.

**Figure 9:**
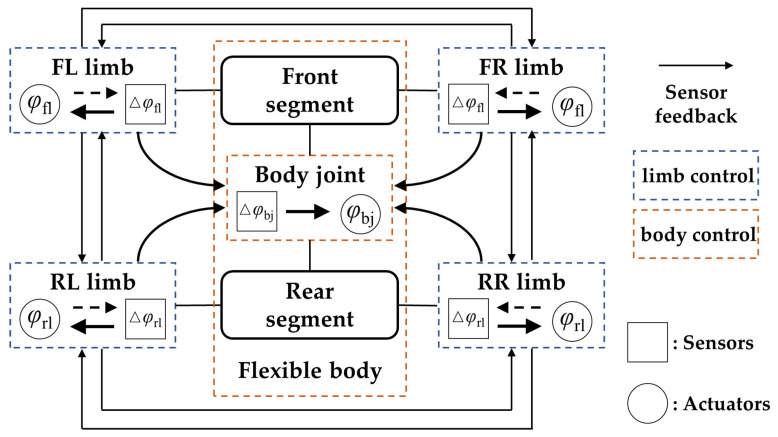
Distributed control with cross-coupled sensory feedback.

Pleurobot [129], based on distributed control, adjusted both the body and limbs’ oscillation phases to synchronize body rotation angles with limb movements, maximizing the motion space of the foot ends. Twister [76] incorporated cross-sensory feedback (as shown in Figure 9), establishing a bidirectional feedback loop between limbs and the body, thereby enhancing the robot’s environmental perception capabilities. The developers of NekoBot [65] asserted that bidirectional feedback between limbs and the body is essential for adaptive coordination and achieving faster speeds in robots. They proposed a minimal body–limb coordination mechanism, whereby robot parts assist each other through sensory feedback mechanisms. This mutual feedback allows each body part to adjust its movements based on the conditions of other parts.

#### 4.2.4. Hierarchical Control

**Figure 10:**
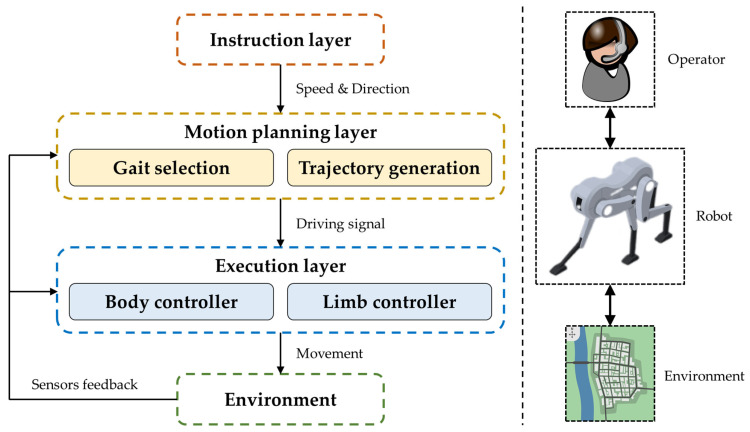
Hierarchical control architecture.

The hierarchical control of flexible-body robots primarily consists of an instruction layer, a motion-planning layer, and an execution layer, as shown in Figure 10. Although some literature expresses hierarchical control differently, the control framework and target functionalities are virtually identical.

The instruction layer primarily receives commands from the operator and conveys motion target parameters, such as speed, direction, and other requirements, to the motion-planning layer. The motion-planning layer is a high-level controller, selecting appropriate gait patterns and generating motion trajectories for the body and limbs based on instructions from the upper layer. These gaits and trajectories can either be selected from a predefined library of gait and trajectory patterns [16] or generated through learning and optimization under constraints based on prior data [126]. The execution layer is a low-level controller that controls each actuator of the robot according to the instructions from the motion-planning layer, realizing the movements of various robot parts. It also implements precise force-position closed-loop control based on sensor feedback. Sensor feedback can also be transmitted to the motion-planning layer, allowing for adjustments to the gait and trajectory, thereby enhancing the robot’s environmental adaptability. In this way, hierarchical control enables the interaction between the operator, the robot, and the environment.

### 4.3. Bionic Control Methods

The following are two typical bionic neural network control methods for robots.

#### 4.3.1. CPG Oscillation Model Control

**Figure 11:**
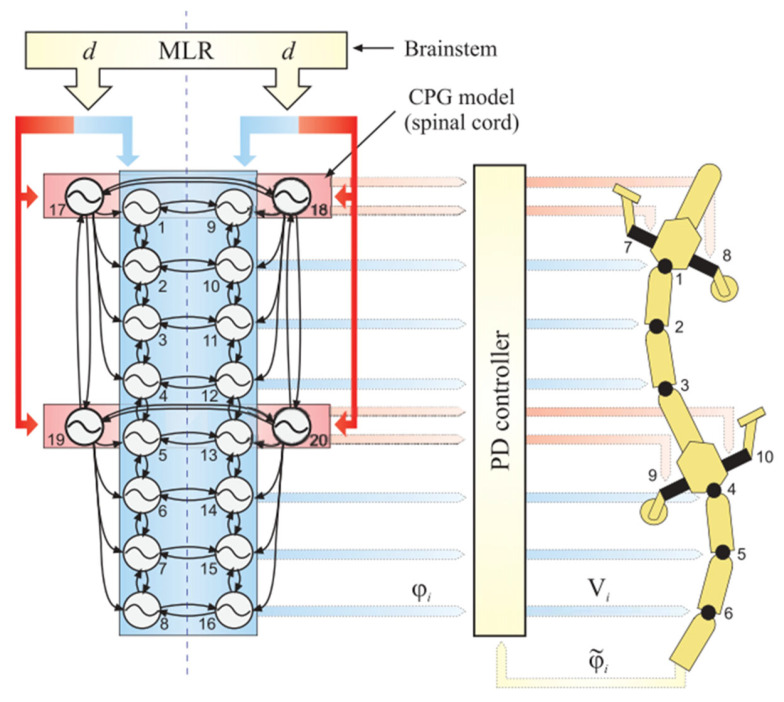
CPG oscillation model control. Figure captured from [40] with permission from AAAS. The meanings of the various parts in the figure can be found in reference [40].

A model based on coupled oscillators was used to investigate the spinal mechanisms underlying the gait transition between walking on land and swimming in Salamandra Robotica I [40], as shown in Figure 11. Coupling between oscillators was achieved through phase differences and connection weights.

CPG oscillation model control combines the characteristics of distributed control and hierarchical control. It consists of a main-body CPG and limb CPGs. The main-body CPG is a double chain composed of 16 oscillators with nearest-neighbor coupling, which is used to drive the spinal motors. The limb CPGs, on the other hand, consist of four oscillators to drive the limb motors. The CPG oscillation model receives left and right drive signals from the MLR (mesencephalic locomotor region). By modifying these two signals, the speed, direction, and gait of the robot can be adjusted.

In subsequent research [131], the CPG oscillation circuit was expanded to achieve richer biomimetic motions, reproducing five behaviors of the salamander on the Salamandra Robotica II: swimming, struggling, underwater forward movement, terrestrial forward movement, and backward movement. The authors of [115] provided an overview of the development process and control of the Salamandra series robots.

#### 4.3.2. Walknet

**Figure 12:**
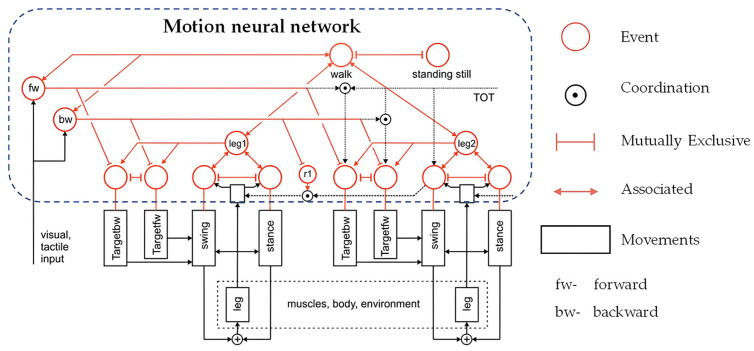
A section of Walknet showing two leg controllers. Figure modified from [133].

Walknet is a bio-inspired multi-legged robot controller that transforms every movement into an event, constructing the motion neural network through the associative or mutually exclusive relationships between these movement events, as shown in Figure 12. Walknet is capable of both controlling individual limbs and coordinating the movements of multiple limbs. Walknet has been successfully applied to Hector [132]. The authors of [133] provided a detailed introduction to the development history of Walknet, which will not be repeated here.

## 5. Summary and Future Challenges

Bionic multi-legged robots with flexible bodies exhibit extremely broad application potential, capable of venturing into the polar regions [127], volcanic craters [57], and even distant extraterrestrial planets [41,134,135,136], assisting humanity in exploring those realms filled with unknowns and challenges. Amid the current wave of interdisciplinary convergence, robotics is intertwining with biology, archaeology, neuroscience, medicine, physics, and other fields to an unprecedented depth, collectively unraveling the mysteries of nature and transforming these discoveries into innovative design principles that drive the continuous optimization of robot and control system designs. This is the core strategy of the emerging field of bionic robotics, which aims to create more efficient and intelligent robotic systems by emulating the wisdom of nature [137].

Bionic robots are not only symbols of technological advancement but also powerful tools for scientific exploration. They can validate biological theoretical models, recreate the morphological characteristics of ancient creatures, and even conduct tests that might be difficult or impossible to perform in traditional biological experiments. On the one hand, biology provides a rich foundation of biological system knowledge for the design of bionic robots; on the other hand, these robots offer controlled and efficient experimental platforms for scientists to verify their theories and hypotheses. Notably, many significant and profound scientific discoveries did not stem directly from observations of nature but were derived from studying phenomena within artificial devices. This is because, compared to the intricate and complex phenomena occurring in nature, artificially constructed systems tend to be more simplified and ordered, making them easier for humans to comprehend and analyze [138]. This unique advantage renders bionic robots an irreplaceable role in scientific exploration.

This article provided a comprehensive review of the structure design, motion, and control of robots with flexible bodies, and concluded that bionic robots continue to confront the following challenges:

- Pursuing more natural patterns in biological morphology and motion

Biomimetic robotics is dedicated to precisely replicating the intricate constructs and motion patterns of animals. This pursuit not only tests design capabilities but also imposes stringent demands on material science, actuation systems, and other hardware components. Specifically, the actuation system, being the heart and soul of a robot’s mobility, often accounts for a significant portion of its overall weight. For instance, in the case of Hector, the actuation system comprised half of the total weight, reaching 55%. For larger bionic robots, the difficulty in designing their actuation systems increases accordingly. The authors of [139] explored the relationship between animal size and locomotor performance and elucidated why the cheetah is an optimal size for speed, providing valuable insights for robot design. Therefore, it is particularly crucial to select appropriate bionic models and determine suitable scaling ratios and parameters for the robots.

To endow robots with smoother and more natural movement, designers continuously integrate more DOFs and compliant elements into their structures, thereby enhancing the robot’s flexibility. However, this requires ensuring the coordinated movement between the limbs and the body.

- Bridging the sensory gap: integrating massive sensors into robotic systems

One of the key challenges facing biomimetic robots is to narrow the sensory gap between machines and animals. While biological systems exhibit remarkable adaptability in complex and dynamic environments, thanks to their rich sensory information and efficient sensorimotor feedback mechanisms, robotic systems struggle to construct comprehensive external environment models and real-time kinematic models. Inspired by this, bionic robots are gradually integrating massive numbers of sensors into their systems to mimic the sensory prowess of living beings. Even without complex internal models, these sensors enable robots to navigate diverse and challenging environments, relying on accurate local environmental modeling and efficient environmental classification capabilities [90].

- Exploring simpler and more efficient control algorithms

Considering the significant increase in control complexity for biomimetic robots, as outlined in the two aspects above, the optimization of control strategies and algorithms has become a sustained focus of attention in this field. The continuous updating and iteration of control algorithms are not only inevitable outcomes of technological progress but also critical driving forces propelling biomimetic robots toward higher levels of development. By continually simplifying algorithm structures and enhancing their efficiency and adaptability, it is hoped to endow biomimetic robots with more intelligent and flexible control capabilities, enabling them to play a significant role in an even broader range of applications.

Ultimately, it is hoped that this article can bring positive inspiration and substantial benefits to the research of bionic robots.

## Figures and Tables

**Table 1:** The characteristics of active spine, compliant spine, single-axis articulation, and multi-axis articulation.

Ways to Achieve Flexible Bodies	Advantages	Disadvantages
Active spine	High flexibility: An active spine can dynamically adjust according to the environment and tasks, providing higher flexibility and adaptability. Better motion performance: It can simulate the movements of animals, enhancing the robot motion ability in complex environments. High control precision: Through a precise control system, an active spine can achieve higher motion precision and coordination, aiding in the completion of complex operational tasks.	More complexity: The design and control system of an active spine are relatively complex, requiring more sensors and control algorithms, which increases the complexity of the robot and the risk of failure. High-energy consumption: Due to the need for continuous energy to drive motors and the control system, an active spine typically consumes more energy. High cost: The manufacturing and maintenance costs of an active spine are higher.
Compliant spine	Simplicity: The design of a compliant spine is relatively simple, reducing the driving element and control systems. Low-energy consumption: Since it does not require driving elements, a compliant spine consumes less energy.	Movement limitations: Due to the lack of active control, a passive spine may perform poorly in certain movement modes, limiting the robot’s motion ability. Poor stability: During rapid movement or on uneven ground, a compliant spine may not provide enough stability, increasing the risk of tipping over.
Single-axis articulation	Simple structure: It is relatively simple, reducing the number of mechanical components. Easy control: Due to the limited range of motion, the control system is relatively straightforward, making it easier to implement basic tasks.	Limited flexibility: Single-axis articulation restricts the robot’s ability to move in multiple directions, resulting in lower adaptability and difficulty in handling complex environments. Movement is limited to a single plane.
Multi-axis articulation	High flexibility: The multi-axis design allows for movement in multiple directions, adapting to complex environments and diverse tasks. Wide range of motion: It can perform more complex motions, such as large-angle turning and bending.	Complex structure: The multi-axis articulated design increases the number of mechanical components. Greater control difficulty: Due to the broad range of motion, the control system becomes more complex, requiring advanced algorithms and sensors to achieve precise movement control.

**Table 2:** Quadruped robots with flexible bodies.

Robot	Ref	Ways to Achieve Flexible Body	Gained DOF	Layout	Active/ Compliant	Bionic Model
BISAM	[61]	Single-axis articulation, Rigid connection	Ry	2 + 2	Active	Ape
Bobcat	[38]	Single-axis articulation, Rigid connection	Ry	2 + 2	Active	Not Given
Canid	[14]	Artificial spine	Ry	2 + 2	Active	Not Given
Charlie	[42]	Stewart platform	Omnidirectional	2 + 2	Active	Ape
Cheetah-Cub-S	[19]	Artificial spine	Rz	2 + 2	Active	Cheetah
Cheetah-I	[1]	Artificial spine	Rz	2 + 2	Compliant	Cheetah
Chigon	[62]	Multi-axis articulation, Rigid connection	Rz, Ry	2 + 2	Active	Salamander
ELIRO-I/II	[2,3]	Single-axis articulation, Rigid connection	Rz	2 + 2	Active	Lizard
Fanari	[30]	Artificial spine	Ry	2 + 2	Compliant	Tiger
GEO-II	[63]	Multi-axis articulation, Rigid connection	Rz, Rx	2 + 2	Active	Not Given
Inu	[15]	Artificial spine	Ry	2 + 2	Active	Not Given
Ken	[39]	Single-axis articulation, Rigid connection	Ry	2 + 2	Compliant	Not Given
Kitty	[10]	Artificial spine	Rz, Ry	2 + 2	Active	Not Given
Laika	[20]	Artificial spine	Rz, Ry, Rx	2 + 2	Active	Not Given
LightDog	[45]	Integrated Elastic material	Rz	2 + 2	Compliant	Dog
LittleApe	[64]	Artificial spine	Rz, Ry	2 + 2	Compliant	Ape
Lynx SV1	[24]	Single-axis articulation, Rigid connection	Ry	2 + 2	Active	Not Given
Lynx SV2 and SV3	[24]	Artificial spine	Ry	2 + 2	Active	Not Given
NekoBot	[65]	Single-axis articulation, Rigid connection	Ry	2 + 2	Active	Not Given
NeRmo	[33]	Artificial spine	Rz, Ry	2 + 2	Active	Rat
Origaker	[5]	Metamorphic		2 + 2	Active	
OroBOT	[66]	Multi-axis articulation, Rigid connection	Rz	2 + 2	Active	Orobates ^1^
OSCILLEX 2/3	[67,68]	Single-axis articulation, Spring connection	Ry, Rx	2 + 2	Compliant	Not Given
Pleurobot	[69]	Multi-axis articulation, Rigid connection	Rz	2 + 2	Active	Salamander
QuaDRoPECS	[18]	Artificial spine	Rz, Ry	2 + 2	Active	Not Given
Renny	[52]	Spheric joint	Rz, Ry, Rx	2 + 2	Active	Cheetah
Salamandra Robotica I ^2^	[36]	Multi-axis articulation, Rigid connection	Rz	2 + 0 + 0 + 2 + 0 + 0 + 0	Active	Salamander
Salamandra Robotica II ^3^	[70]	Multi-axis articulation, Rigid connection	Rz	0 + 2 + 0 + 0 + 0 + 2 + 0 + 0 + 0	Active	Salamander
Serval	[37]	Multi-axis articulation, Spring connection	Rz, Ry	2 + 2	Active	Dog
Slalom	[71]	Multi-axis articulation, Rigid connection	Rz	2 + 2	Compliant	Gecko
SQBot	[72]	Single-axis articulation, Spring connection	Ry	2 + 2	Compliant	Cheetah
SQuRo	[73]	Multi-axis articulation, Rigid connection	Rz	2 + 2	Active	Rat
SQuRo-S	[74]	Artificial spine	Rz, Ry, Rx	2 + 2	Active	Rat
Stoch 2	[7]	Multi-axis articulation, Rigid connection	Ry	2 + 2	Active	Cheetah
Sugoi-Neco	[75]	Single-axis articulation, Spring connection	Ry	2 + 2	Compliant	Cat
Tiger	[29]	Artificial spine	Ry	2 + 2	Compliant	Tiger
Transleg	[12]	Artificial spine	Rz, Ry	2 + 2	Active	Not Given
Twister	[76]	Single-axis articulation, Rigid connection	Rz	2 + 2	Active	Not Given
Unnamed	[46]	Integrated Elastic material	Rz	2 + 2	Active	Lizard
Unnamed	[53]	Octagon-origami	Rz, X	2 + 2	Active	Inchworm
Unnamed	[47]	Piezoelectric material	Ry	2 + 2	Active	Cockroach
Unnamed	[28]	Artificial spine	Rz	2 + 2	Compliant	Not Given
Unnamed	[54]	Soft structure	Ry	2 + 2	Compliant	Cheetah
Unnamed	[17]	Artificial spine	Rz	2 + 2	Active	Dog
Unnamed	[22]	Artificial spine	Rz, Ry, Rx	2 + 2	Active	Cat
Unnamed	[21]	Artificial spine	Rz, Ry	2 + 2	Active	Not Given
Unnamed	[77]	Single-axis articulation, Rigid connection	Rz	2 + 2	Compliant	Gecko
Unnamed	[78]	Single-axis articulation, Rigid connection	Rz	2 + 2	Active	Gecko
Unnamed	[79]	Single-axis articulation, Rigid connection	Rz	2 + 2	Active	Salamander
Unnamed	[41]	Multi-axis articulation, Spring connection	Rz, Ry, Rx	2 + 2	Active	Chameleon
WR Series Robots (1–5 and 5M)	[80,81,82,83,84]	Multi-axis articulation, Rigid connection	Rz, Ry	2 + 2	Active	Rat
Yat-sen Lion	[85]	Multi-axis articulation, Rigid connection	Rz, Ry	2 + 2	Active	Not Given

^1^ Orobates is an extinct animal. ^2^ Salamandra Robotica I, as shown in Figure 5. It is composed of seven segments, with two legs on each of the first and fourth segments, the second and third segments serving as the flexible body and multi-axis articulation connected, while the fifth to seventh segments function as the tail of the robot. ^3^ Salamandra Robotica II is composed of nine segments, with two legs on each of the second and sixth segments. The first segment serves as the head of the robot, the third to fifth segments serve as the body, and the seventh to ninth segments serve as the tail.

**Table 3:** Multi-legged robots ^1^ with flexible bodies.

Robot	Ref	Ways to Achieve Body Flexible	Gained DOF	Layout	Active/ Compliant	Bionic Model
AMOS Series Robots (II and WD06)	[4,86]	Single-axis articulation, Rigid connection	Ry	2 + 4	Active	Cockroach
CkBot	[87]	Multi-axis articulation, Rigid connection	Rz, Rx	2 + 2 + 2 + ……	Active	Centipede
Dante II	[57]	Reciprocating Mechanism	Rz, X	3 + 3	Active	
Hector	[8]	2 DOFs Spindle Joint	Rz, Ry, X	2 + 2 + 2		Stick insects
IOAN	[88]	Multi-axis articulation, Rigid connection	Ry, Rx	2 + 2 + 2	Active	Not Given
MELCRAB-2	[6]	Reciprocating Mechanism	Rz, X	3 + 3	Active	
ModPod	[89]	Multi-axis articulation, Rigid connection	Rz, Ry	2 + 2 + 2	Active	Cockroach
Octopod Robot	[34]	Single-axis articulation, Rigid connection	Ry	4 + 4	Active	
Octopod Robot	[55]	Bricard linkages	Rz, Ry	4 + 4	Active	
ParaWalker	[43]	Stewart Platform	Omnidirectional	3 + 3	Active	
SCORPION-III/IV	[90]	Soft structure (rubber)	Rz, Ry, Rx	2 + 4 + 2	Compliant	Scorpion
SpaceClimber	[9]	Single-axis articulation, Rigid connection	Ry	2 + 4	Active	Not Given
Unnamed	[58]	Reciprocating Mechanism	Rz, Y, X	3 + 3	Active	
Unnamed	[91]	Single-axis articulation, Rigid connection	Rz	2 + 2 + 2 + 2 + 2 + 2	Active	Centipede
Unnamed	[92]	Sarrus linkages	Rz, X	2 + 2 + 2 + ……	Compliant	Centipede
Unnamed	[93]	Spheric joint	Rz, Ry, Rx	2 + 2 + 2 + ……	Compliant	Centipede
Unnamed	[94]	Single-axis articulation, Rigid connection	Ry	2 + 2 + 2 + 2 + 2 + 2 + 2	Active	Sea roaches
Whegs^TM^ Series Robots (II, IV, and Climbing Mini-Whegs^TM^)	[95,96,97]	Single-axis articulation, Rigid connection	Ry	2 + 4	Active	Cockroach

^1^ Multi-legged robots refer to robots with more than four legs in Table 3.

**Table 4:** The impact of each DOF on the robot’s motions.

DOF	Motion of Flexible Body	Benefits to the Robot’s Motions
Rz	Yaw: Bending in the horizontal plane	Crawling and trotting, larger step length, higher speed, and wider workspace Smaller turning radius Better stability
Ry	Pitch: Bending in the sagittal plane	Bounding and galloping, larger step length, higher speed, and wider workspace Better obstacle-crossing capability Higher energy efficiency (for robots with artificial spines)
Rx	Roll: Bending in the frontal plane	Better terrain adaptability and traversability Swing leg
X	Body extension	Moving forward (for inchworm-inspired robots and double-support-frame robots)

## Data Availability

Not applicable.
